# Development and psychometric properties of a questionnaire to measure drug users’ attitudes toward methadone maintenance treatment (DUAMMT) in Iran

**DOI:** 10.1186/s12889-017-4911-6

**Published:** 2017-11-28

**Authors:** Maryam Khazaee-Pool, Zohreh Arefi, Daem Roshani, Tahereh Pashaei, Koen Ponnet

**Affiliations:** 1 Department of Health Education and Promotion, School of Public Health, Zanjan University of Medical Sciences, Zanjan, Iran; 2 Department of public health, faculty of health, Kurdistan University of medical sciences, Sanandaj, Iran; 3 Environmental Health Research Center, Kurdistan University of Medical Sciences, Sanandaj, Iran; 4 Department of Communication Studies, IMEC-MICT, Ghent University, Ghent, Belgium

**Keywords:** Attitude, Methadone maintenance, Instrument, Psychometrics, DUAMMT

## Abstract

**Background:**

Assessing drug users’ attitudes towards different kinds of addiction treatment is necessary to design tailored strategies. The aim of the present study is to develop and examine the psychometric properties of a new scale, called the DUAMMT, for assessing drug users’ attitudes toward methadone maintenance treatment in Iran.

**Methods:**

A multi-phase development method was applied in developing an instrument from February to December 2016. The item generation and scale development were performed through literature review, a qualitative approach, and interviews with an expert panel. Then, the psychometric properties of the scale were evaluated by means of cross-sectional studies with drug users. We performed an exploratory factor analysis, a confirmatory factor analysis, and item-scale correlations; and we tested the internal consistency of the scale. Furthermore, test-retest reliability was evaluated among an Iranian sample of drug users.

**Results:**

The mean age of participants was 34.12 years. The exploratory factor analysis revealed four factors (perceived barriers, perceived concerns, methadone side effects, and perceived positive effects) containing 17 items that jointly accounted for 60.53% of the observed variance. The confirmatory factor analysis showed a model with appropriate fitness for the data. The Cronbach’s alpha coefficient for the subscales ranged from .70 to .79. The intra-class correlation coefficient (ICC) ranged from .774 to .970, which is well above the acceptable threshold.

**Conclusions:**

The findings of the present study suggest that the DUAMMT is a valid and reliable instrument to measure drug users’ attitudes toward methadone maintenance treatment. The DUAMMT can be applied at the start of treatment so that clinical intervention can be targeted to promote retention in treatment.

## Background

The International Classification of Diseases (ICD-10) has classified opioid dependence as a chronic and relapsing disorder [1]. Opioid use disorder has been a main contributor to comorbidity and premature mortality caused by overdose and blood-borne infections, such as human immunodeficiency virus (HIV) and hepatitis [2–6]. In addition, opioid dependence was responsible for 51,000 deaths throughout the world in 2013 [7] and also accounted for the greatest proportion of universal disability-adjusted life years (DALYs) attributed to drug dependence, i.e., 9.2 million DALYs in 2010 [8]. More concerns may be raised considering the fact that DALYs attributed to opioid use disorder increased during the time period of 1990–2010 [8]. Aside from physical harm, previous studies have also shown that people with opioid use disorders have higher risks of panic disorders, social phobia, agoraphobia, low self-reported health, lifetime anxiety, and mood disorders [2, 9, 10].

Methadone, which is a synthetic opioid with potent analgesic effects, was initiated in Germany during World War II and was prescribed as a painkiller. In 1964, however, the first “methadone program” was established by Dole and Nyswalder (1965) in order to treat heroin dependence [11, 12]. Methadone maintenance treatment (MMT) is an opioid replacement therapy (ORT); taking stable daily doses of methadone in the long term has been proven to alleviate the uncomfortable withdrawal symptoms of opioid abstinence, reduce opioid craving, and block opioid euphoria [6, 11, 12]. Although opioid replacement therapies have not been limited to methadone maintenance treatments in contemporary decades, MMT has been widely accepted as one of the best evidence-based medication-assisted therapies for chronic opioid dependence and can be considered a harm reduction strategy [6, 12–14]. Methadone has a positive influence on public health and security as well as human capital and social productivity [15–17]. There is evidence that in low- and middle-income countries where there is a lack of treatment programs, the expansion of MMT programs might lead to savings in social and health expenditures [5, 13, 18]. Meta-analysis of different data found that MMT is highly efficient in reducing heroin dependence [19], reducing risky behavior related to HIV transmission [20], reducing overdose-related deaths [21], and reducing crime rate [22].

Although MMT programs are one of the most important treatment strategies to reduce individual and public harm associated with opioid use, and despite the central role of methadone therapy in harm reduction approaches to opioid use in Iran as well as many other countries, previous studies have pointed out that a large proportion of eligible patients refuse to participate in this treatment program [4, 16, 23–25]. There are some factors that prevent the tendency of patients to use this treatment, such as lack of access and high cost. Evidence has shown that some other items, such as attitudes and beliefs of patients regarding methadone treatment, can affect their acceptance of this treatment [26]. Positive attitudes toward methadone treatment have been related to retention in treatment [27].

Thus, assessing drug users’ attitudes toward different kinds of addiction treatment may be necessary to design more effective treatment plans. High prevalence of illicit drug use [28]; being close to major illicit drug production regions in Afghanistan [29]; social, cultural, and economic special conditions [30]; and huge problems regarding drug treatment [31] are all reasons the development of instruments related to addiction treatment in Iran has priority. A United Nations Office on Drug and Crimes (UNODC) report indicated that more than 80% of the recognized drug treatment seekers in Iran were primarily people with opioid use disorders [32]. Although many Iranian researchers have attempted to develop instruments for addiction treatment [33–36], none of them focused on respondents’ attitudes toward methadone. Thus, the aim of the present study is to develop and examine the psychometric properties of a new scale, called the DUAMMT, for assessing Iranian drug users’ attitudes toward methadone maintenance treatment.

## Methods

### Research design

This study was approved by the Ethics Committee of Kurdistan University of Medical Sciences [Grant number 14/23311], and all patients completed informed written consent. The study was conducted in two phases. Firstly, item generation and scale development were performed by applying three approaches: a literature review, a qualitative method approach, and interviews with an expert panel. In the second phase, the psychometric properties of the scale were evaluated by means of cross-sectional studies with drug users. We performed exploratory factor analysis, confirmatory factor analysis, and item-scale correlation, and we assessed the internal consistency of the scale. Furthermore, test-retest reliability was evaluated among an independent sample of 30 drug users. Table 1 provides the descriptive characteristics of the participants from the two phases.

**Table 1:** Characteristics of the study sample

		EFA sample (*n* = 200)	CFA sample (n = 120)	Test-retest sample (*n* = 30)
Age (years)				
	Mean	34.12 ± 8.57	36.12 ± 9.43	27.11 ± 6.11
	Range	21–70	18–75	22–48
Employment status				
(n / %)	Unemployed	44 (22%)	42 (21%)	11 (36%)
	Employed full time	156 (78%)	78 (39%)	19 (63%)
Educational level				
(n / %)	Primary	67 (33%)	53 (44.1%)	7 (23%)
	Secondary	101 (50%)	42 (35%)	15 (50%)
	Higher	32 (16%)	26 (21.6%)	8 (26%)
Marital status				
(n / %)	Single/divorced/widowed	87 (43%)	43 (35.8%)	13 (43.3%)
	Married	113 (56.5%)	77 (64.1%)	17 (56.6%)

### Phase 1: Item generation and scale development phase

In this phase, we aimed to develop an instrument to measure drug users’ attitudes toward MMT. Two methods were applied to develop an item pool in the present study:

First, a qualitative study was designed to explore the drug users’ attitudes toward MMT. For the purpose of this phase, 12 individual interviews were conducted among a sample of drug users. Patients were recruited from drop-in centers (DICs) and MMT centers in Sanandaj, Iran. The DICs are run by local nongovernmental organizations offering therapies and psychosocial support and facilitating self-help groups. Maximum variation sampling was used in this phase, meaning that we recruited participants with different sociodemographic characteristics so that they complement each other’s viewpoints in experiencing methadone. In order to obtain maximum variation, patients engaging in various types of drug use were chosen from different ages and socioeconomic backgrounds. The descriptive characteristics of patients are shown in Table 1. In-depth individual interviews delivered a condition for us to talk about patients’ beliefs about methadone treatment and, as a result, to recognize the level of their attitudes. Patients had different levels of education.

The interviews were initiated by defining maintenance treatment and applying a semi-structured inventory that started with an open-ended question: “What is your opinion about methadone treatment?” Then, based on the answers from the patients, several questions were asked to promote discussion. All discussions were recorded, and we wrote our analytic concepts in a memo text.

All patients were informed about the aim of the study, and they filled in the informed consent. The interviews were held in the DIC and MMT centers, and all discussions were tape-recorded. Data saturation was achieved after 12 individual interviews. Afterward, we applied an inductive method to analyze the recorded discussions. Inductive content analysis was applied to detect themes by studying the raw data of the interviews through continuous comparison [37]. Clear procedures were used to draw conclusions from the interviews in order to ensure credibility. For transferability, we provided rich explanations that can be applied by other researchers to other situations and backgrounds. Furthermore, in order to ensure conformability, we checked the internal coherence of the results [38].

Thereafter, experts were asked, “What are the most effective treatments for addiction and relapse prevention in people with opioid use disorders? Why do you consider these important? And why do you think the other treatments for drug users are not as important as your selected approaches?”

In the end, all data obtained from qualitative research and interviews with experts were cross-checked, and based on the three approaches, 30 items in Farsi were created for an initial scale. Each item was rated on a 5-point Likert scale ranging from 1 (strongly agree) to 5 (strongly disagree). Subsequently, content and face validity were evaluated.

### Content validity

In this study, both qualitative and quantitative content validity (content validity index/ratio) were assessed. In the qualitative stage, a scientific panel of 10 experts (including health educators, psychologists, and addiction therapists) evaluated the initial scale. They evaluated the grammar, wording, and scaling of each item. To assess the quantitative content validity, both the content validity index (CVI) and content validity ratio (CVR) were calculated. The simplicity, accuracy, and clarity of each item were measured by the CVI [39, 40]. In order to calculate the CVI, a 4-point Likert-type ordinal scale was applied by the expert panel. The answers were rated between 1 (not relevant, not clear, and not simple) and 4 (very relevant, very clear, and very simple). The CVI was assessed as the proportion of items that received a rating of 3 or 4 by the experts [41]. A CVI score lower than .80 was not acceptable [42]. The essentiality of the items was tested by the CVR. Each item was scored by the expert panel as 1 (essential), 2 (useful but not essential), or 3 (not essential) [41]. Then, based on the Lawshe Table [43], items with a CVR score of 0.62 or above were considered to be acceptable and were retained.

In the quantitative stage, items with a CVR and CVI less than .62 and .82, respectively, were deleted.

In total, 8 items were deleted, resulting in a 22-item pool. Furthermore, the expert panel revised the scale with regard to grammar, wording, and item allocation. For example, the sentence “Methadone treatment does not reduce return to reusing drug” was changed to “Methadone does not have an effect on the prevention of relapse.” The 22-item pool remained in the analyses below and consisted of positively worded and negatively worded statements with five response options: 1 = totally disagree, 2 = disagree, 3 = neither disagree nor agree, 4 = agree, and 5 = totally agree.

### Face validity

In this step, both qualitative and quantitative approaches were used to assess face validity. A group of drug users (*n* = 10) were asked to evaluate each item of the scale and to indicate if they felt ambiguity or difficulty in answering the Iranian version of the DUAMMT questionnaire. Based on the respondents’ perspectives, the ambiguous items were adapted. In the quantitative phase, the impact score (frequency × importance) was assessed to show the percentage of drug users who identified each item as important or somewhat important on a 5-point Likert scale. Items were considered to be inappropriate if they had an impact score less than 1.5 (which matches a mean frequency of 50% and a mean importance of three on the 5-point Likert scale) [44]. Overall, three items had an impact score less than 1.5 and were deleted. The range of the impact score for the remaining 19 items was from 1.7 to 5. The first form of the questionnaire containing 19 items was established for the next phase of psychometric evaluation.

### Phase 2: Psychometric phase

#### The main study and the data collection

In order to assess the psychometric properties of the DUAMMT questionnaire in a wider setting, a cross-sectional study was designed to be carried out in Sanandaj, Iran, from February to December 2016. A simple random sampling method was applied. Firstly, four DIC and MMT centers were randomly selected among DIC and MMT centers in Sanandaj, Iran. patients who visited DIC and MMT centers were entered into the study if they were male patients with substance abuse referred to harm reduction centers, met the diagnostic criteria for substance dependence disorder based on the DSM-IV, were literate, and wanted to take part in the study. After the main investigator provided an explanation about the aim of the study, patients who agreed to take part in this study completed the DUAMMT questionnaire. In addition, the demographic characteristics of patients including age, educational level, employment status, marital status, and type of drug use were also collected. In order to collect data, educated investigators conducted face-to-face interviews.

### Statistical analysis

Several statistical methods were applied to test the psychometric properties of the DUAMMT scale. These are presented as follows.

### Validity

#### Construct validity

After the item analysis, the 19 remaining items were considered to estimate the construct validity using exploratory factor analysis (EFA), confirmatory factor analysis (CFA), and item-scale correlation.

##### Exploratory factor analysis

EFA was performed to identify the main factors of the scale. The sample size was estimated a priori. As recommended by Gable and Wolf, a sample of five to ten patients per item is required to ensure a conceptually clear factor structure for EFA [45]. The preferred maximum required sample size was thus determined to be 200 drug users. These patients were recruited from the DIC and MMT centers. The main factors of the scale were extracted by performing EFA, applying the principal component analysis (PCA) with varimax rotation. The Kaiser-Meyer-Olkin (KMO) test and Bartlett’s test of sphericity were applied to assess the adequacy of the sample for the factor analysis [46]. In order to extract the factors, a factor with an eigenvalue above 1 was considered significant. Additionally, a scree plot was used to specify the number of factors. Factor loadings equal to or greater than .40 were considered acceptable [47].

##### Confirmatory factor analysis (CFA)

A CFA was performed in order to assess the fit of the model. Considering the possible attrition related to test-retest analysis, a separate sample of 120 drug users was planned to be recruited from harm reduction, MMT, and DIC centers. Assigning seven patients to each item, a sample size of 120 was estimated [48]. The model fit was evaluated using multiple fit indices. As suggested, several fit indices measuring relative chi-square (χ2/df), comparative fit index (CFI), goodness of fit index (GFI), non-normed fit index (NNFI), normed fit index (NFI), root mean square error of approximation (RMSEA), and standardized root mean square residual (SRMR) were taken into account [49, 50]. Relative chi-square is the chi-square ratio to degrees-of-freedom, and it is suggested that a value less than 3 indicates an acceptable fit [51]. The values of GFI, CFI, NNFI, and NF range from 0 to 1, but values equal to 0.90 or above are commonly indicated as acceptable model fits [52]. An RMSEA value between .08 and .10 indicates an average fit, and a value below .08 shows a good fit. Values below .05 indicate a good fit for SRMR, but values between .05 and .08, and between .08 and .10, indicate a close fit or are acceptable, respectively [53].

##### Item-scale correlation

Finally, item-scale correlations were calculated in order to assess the degree to which each item was correlated to its subscale by use of the Spearman correlation coefficient. We expected that, for each subscale of the DUAMMT, the item scores of the subscale (e.g., perceived barriers) would correlate more with the total score of the respective subscale (e.g., perceived barriers) rather than the total score of other subscales (e.g., perceived side effects). Correlation values between 0 and .20 are considered poor; between .21 and .40, fair; between .41 and .60, good; between .61 and .80, very good; and above .81, excellent [54].

#### Reliability

##### Internal consistency

The internal consistency of the DUAMMT questionnaire was assessed by calculating the Cronbach’s’ alpha coefficient of the whole scale and each dimension of the DUAMMT questionnaire. Alpha values equal to .70 or higher were considered acceptable [54, 55].

##### Test-retest

A subsample of drug users (*n* = 30) filled out the DUAMMT questionnaire twice, with a 2-week interval, in order to examine the stability of the questionnaire by estimating the intra-class correlation coefficient (ICC). ICC values of .40 or above are considered acceptable [55]. All statistical analyses, except CFA, were performed using SPSS 18.0 [56]. The CFA was performed using LISREL 8.80 [57].

##### DUAMMT questionnaire

The final version of the DUAMMT questionnaire is shown in Appendix 1. Each item is rated on a five-point response scale. Four items were negatively worded (items 2, 11, 13, and 15) and have to be reverse scored [Appendix 2].

## Results

### Construct validity

#### Exploratory factor analysis

**Fig. 1:**
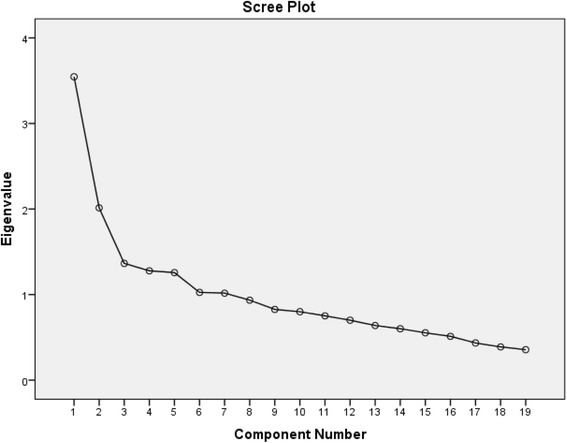
Scree plot for determining factors of the designed instrument

The measured KMO was .742, and the Bartlett’s test of sphericity was significant (χ2 = 644.03, *p* < .001), indicating adequacy of the sample for EFA. Initially, for the 19-item scale, seven factors showed eigenvalues above 1.0, explaining the 60.53% variance. Additionally, the scree plot showed a 4-factor solution (Fig. 1). This 4-factor solution was explored by repeatedly assessing the item performance by eliminating the items in a step-by-step process. After removing the items with factor loadings below .40, a final factor solution was obtained that consisted of a 17-item questionnaire loading on four distinct constructs. These constructs jointly accounted for 60.53% of the observed variance.

As presented in Table 2, four factors were found: Factor 1 (perceived barriers toward methadone treatment) included 7 items (items 11, 12, 13, 14, 15, 16, and 17), factor 2 (perceived side effects) included 4 items (items 1, 2, 3, and 4), factor 3 (perceived concerns) included 3 items (items 5, 6, and 7), and factor 4 (perceived positive effects) included 3 items (items 8, 9, and 10). Refer to Appendix 1 for the items of the DUAMMT.

**Table 2:** Exploratory factor analysis of the DUAMMT (n = 200)

	Factor 1	Factor 2	Factor 3	Factor 4
Q12. Methadone treatment is long term.	**.762**	.079	.075	−.190
Q16. I do not receive adequate counseling at the Methadone Therapy Center.	**.699**	.042	.253	.010
Q13. Methadone does not have an effect on the prevention of relapse.	**.690**	−.133	.139	−.209
Q15. Methadone treatment centers are not available enough.	**.633**	.023	.421	.110
Q14. Daily clinic visits for methadone are hard for me.	**.507**	.115	.056	.383
Q17. Methadone treatment is costly.	**.424**	.081	.261	.400
Q11. Methadone does not help addiction treatment.	**.406**	.324	−.096	.326
Q18. Methadone reduces contact with drug dealers	.281	.225	.210	.338
Q19. Methadone reduces clashes with the police.	.344	−.241	.236	305
Q1. Methadone is addictive.	.107	**.669**	−.125	−.069
Q3. Methadone decreases my sexual activity.	.060	**.608**	.078	.023
Q2. Methadone makes me sleepy.	−.196	**.475**	.169	.096
Q4. Methadone has negative effects on my health (constipation, liver problems, heart pain).	−.018	**.459**	.001	−.065
Q6. Daily clinic visits for methadone have a negative effect on my family relationships.	.241	−.140	**.695**	.086
Q7. I’m scared to see one of my relatives in a methadone treatment center.	.202	−.038	**.677**	.173
Q5. Daily clinic visits for methadone threaten my family life and my job.	−.109	.331	**.520**	−.248
Q10. Methadone makes me feel euphoric.	−.102	.082	.173	**.616**
Q8. Methadone reduces the temptation to consume drugs.	.153	.312	.030	**.529**
Q9. I’ve had more clean days with methadone.	−.065	.352	.293	**.523**

Note. Figures in bold represent factor loadings equal to or above .40

#### Confirmatory factor analysis

**Fig. 2:**
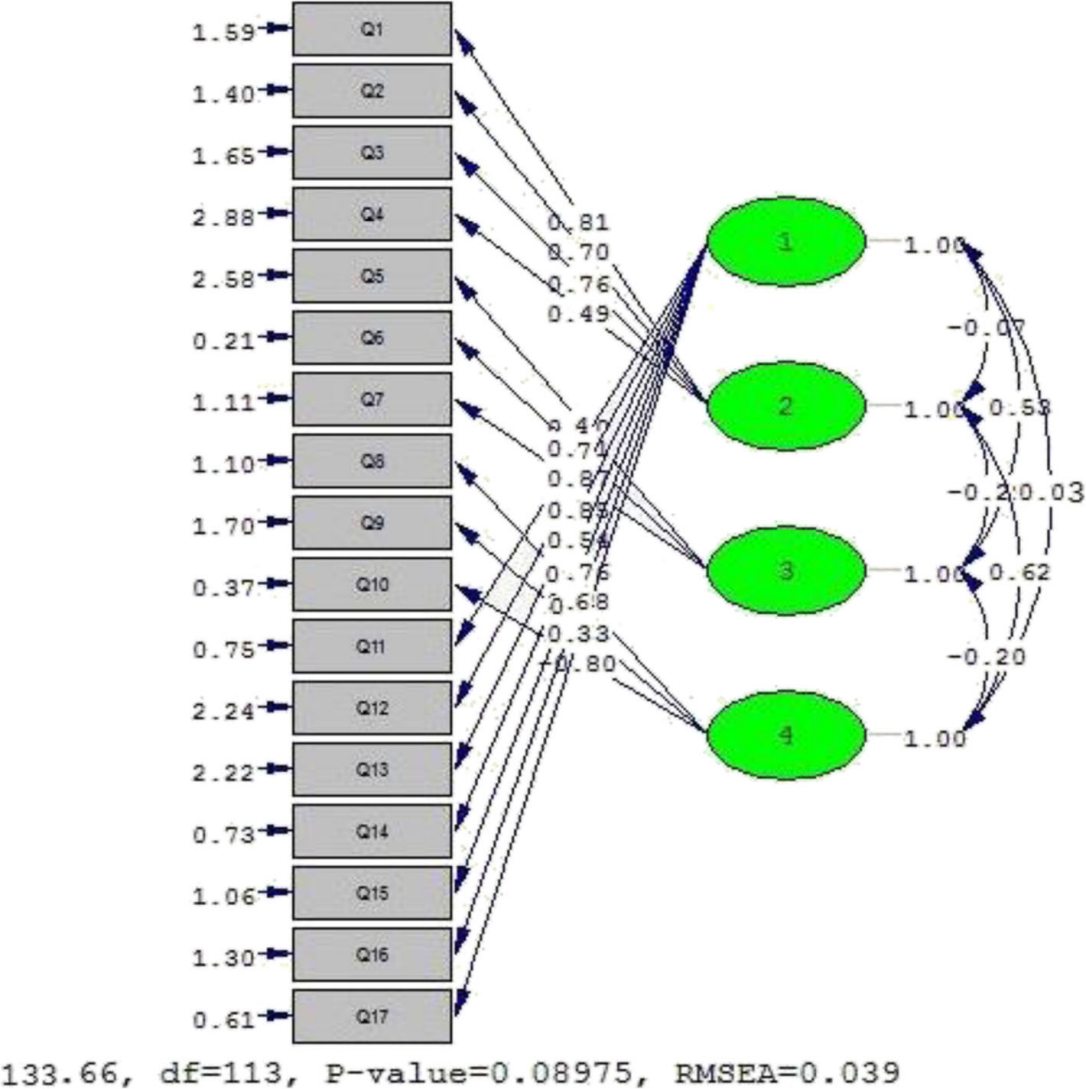
A four-factor model for the questionnaire obtained through confirmatory factor analysis (*n* = 120)

CFA on the 17-item DUAMMT questionnaire was conducted to test the fitness of the model obtained from the EFA. Figure 2 shows the best model fit for the DUAMMT questionnaire. Covariance matrices were used, and fit indices were calculated. All fit indices proved to be acceptable. The relative chi-square (χ2/df) was equal to 2.04 (*p* < .001). The RMSEA of the model was .039 (90% CI = .001–.063), and the SRMR was .030. All comparative indices of the model, including GFI, AGFI, CFI, NNFI, and NFI, were more than .80 (.88, .84, .92, .90, and .80, respectively).

#### Item-scale correlation

Table 3 presents the item-scale correlation for the DUAMMT questionnaire. As can be seen, all coefficients are higher than .20, and most of them are higher than 0.40. Perceived barriers and perceived positive effects had the lowest and the highest item-scale correlation, respectively.

**Table 3:** Item-scale correlation matrix for the four DUAMMT constructs (n = 200)

DUAMMT Dimensions				
	PB	PSE	PC	PPE
PB (item number)				
Item 11	***.340***	.049	.015	−.018
Item 12	***.419***	−.036	.127	.120
Item 13	***.440***	−.012	−.102	.205
Item 14	***.628***	−.016	.075	.071
Item 15	***.781***	.040	.218	.044
Item 16	***.754***	−.048	.248	.038
Item 17	***222***	−.029	.078	−.087
MSE (item number)				
Item 1	−.027	***.631***	−.102	.251
Item 2	−.016	***.603***	−.104	.266
Item 3	−.083	***.604***	.001	.094
Item 4	.085	***.621***	−.086	.206
PC (item number)				
Item 5	−.160	.069	***.642***	.113
I tem 6	.364	−.183	***.708***	−.189
Item 7	.246	−.179	***.703***	.057
PPE (item number)				
Item 8	.083	.303	−.025	***.829***
Item 9	.004	.243	.046	***.767***
Item 10	.094	−.056	−.013	***.089***

Note^1^. *PB* Perceived barriers, *PSE* Perceived side effects, *PC* Perceived concerns, *PPE* Perceived positive effects

Note^2^. The bold and italicize data reflect higher correlations for the four DUAMMT dimensions. All coefficients are higher than .20

### Reliability

In order to measure the reliability, the Cronbach’s alpha was calculated separately for the DUAMMT and each factor of the DUAMMT. The Cronbach’s alpha coefficient for the DUAMMT was .93 and ranged from .70 to .79 for its subscales, which is well above the acceptable threshold. Therefore, no items of the scale were omitted in this phase. In addition, test-retest analysis was conducted to test the stability of the DUAMMT questionnaire. The results indicated satisfactory results. ICC was .94 (good to excellent) for the DUAMMT and ranged from .774 to .970 for the subscales of the DUAMMT, lending support for the stability of the scale. The results are presented in Table 4.

**Table 4:** Measures of internal consistency and stability

Factor	Name	Number of items	Cronbach’s alpha (*n* = 200)	ICC (*n* = 30)
1	Perceived barriers	7 items (11–17)	.788	.844
2	Perceived side effects	4 items (1–4)	.710	.774
3	Perceived concerns	3 items (5–7)	.704	.784
4	Perceived positive effects	3 items (8–10)	.725	.970
Total		17 items	.932	.938

## Discussion

Because there is not a questionnaire to measure attitudes toward methadone in Iran, the present study as initial research described the development and psychometric properties of the questionnaire for assessing the attitudes toward methadone maintenance treatment in Iran. The results demonstrated that the final 17-item DUAMMT questionnaire is a robust, valid, and reliable questionnaire that comprises four subscales (perceived barriers, perceived concerns, methadone side effects, and perceived positive effects).

Overall literature review showed similar studies developing scales for assessing attitudes toward methadone. For instance, the 36-item Brown questionnaire that assesses attitude toward methadone contains two factors (barriers and benefits of methadone) [58]. Similarly, Kayman et al. developed a 14-item questionnaire to measure beliefs about methadone that contained four constructs: benefits of MMT, treatment-related barriers, reduction of crime, and feelings about leaving the methadone program [59]. In addition, Caplehorn developed a 53-item questionnaire to measure attitudes toward addiction and methadone, which included knowledge about methadone maintenance, disapproval of drug use, abstinent-orientation, attitude toward methadone, and attitude toward illegal drugs. In Caplehorn’s study, attitudes about methadone were not specifically investigated in their questionnaire; they only considered attitudes about illegal drugs [60]. Also, Schwartz and colleagues assessed attitudes toward methadone by using the attitudes toward methadone scale that contains 28 items related to perceptions of methadone potential helpfulness, negative physical and cognitive effects associated with methadone, and the perceived purpose of methadone treatment.

It is noteworthy that so far, none of these tools have been translated or used in any research in Iran. Other similar tools are more focused on barriers and perceived benefits, but in the DUAMMT, perceived barriers are explained in detail, and methadone side effects have been investigated in detail. Most of the participants in the qualitative study indicated methadone side effects.

The items of the DUAMMT were developed using a qualitative study. Thereafter, we conducted both exploratory and factor analyses; the results showed that the structure of the questionnaire was good. EFA showed that the total variance of the questionnaire was 60%, and the CFA indicated that the factor structure of the questionnaire was suitable. In this study, the χ^2^/df ratio was 1.43, the GFI for the model was 0.001, the SRMR was 0.80, and the NNFI was 0.92. These results indicate that the model was a very good fit for our data.

According to EFA, four latent factors were extracted. These subscales were named by considering the concepts and after several meetings with expert panel members. In the present study, we also applied CFA, which is superior compared to analyses of other studies, such as those used in the study by Kayman and colleagues [59] or Brown and colleagues [58].

The internal consistency of the final instrument as assessed by the Cronbach’s alpha and the test-retest coefficient was found to be .70 and .93, respectively, indicating acceptable reliability and homogeneity of the items of the DUAMMT. Compared to the Brown questionnaire, the Cronbach’s alpha of the DUAMMT was higher at 0.89 vs 0.68. The Caplehorn scale calculated only the overall Cronbach’s alpha, with a value of 0.89 [60]. The reliability of the present questionnaire was also higher than that of the Kayman et al. [59] questionnaire (0.60), indicating the fundamental reliability of this tool among the patients struggling with drug abuse.

The DUAMMT is able to identify attitudes toward methadone as well as predict retention among patients. Kayman and colleagues, utilizing an abbreviated version of the methadone scale, found that attitudes toward methadone predict retention in methadone treatment [59]. Based on the results, the validity proved to be good, as well as the reliability and stability of the questionnaire. There is a lack of appropriate tools to measure attitudes toward methadone in Iran. This questionnaire can be used as a standard questionnaire in future studies.

The present study, however, has some limitations. First, with regard to the sampling, we only interviewed drug users who were treated in MMT centers in Sanandaj. Because these patients are culturally homogeneous, their viewpoints may not be generalized to the views of patients treated in other cultures. Consequently, it might be interesting for future studies to test the reliability and validity of the DUAMMT in a sample of drug users from different cultural backgrounds and areas. Second, regarding the sampling, 22% of the patients in the present study were unemployed and 100% were men. In future studies, it would be necessary to examine the psychometric properties of the DUAMMT in patients from both urban and rural areas with different levels of education, employment status, and economic status. Third, the DUAMMT was developed by only using samples of men, and was tested among men, as a result, it may not be representative of women with opioid use disorders. Future studies should examine its validity and reliability among women as well. Furthermore, in this study, we did not test how the DUAMMT is associated with similar scales. However, one of the strengths of the study is that two separate samples were recruited for the EFA and CFA. Attitude toward methadone treatment is related to retention in treatment. Also, long-term retention in treatment has a favorable outcome for both patients and society.

## Conclusions

Generally, the findings of the present study suggest that the DUAMMT is a valid and reliable instrument to measure the attitudes towards MMT among male opioid users in Iran. Further studies in different populations, and particularly with women, are recommended to establish stronger psychometric properties for the questionnaire. Such studies can enhance the tailoring of appropriate MMT to optimize successful outcomes among people with opioid use disorders.

## Appendix 1

**PSE: Perceived side effects.**

Q1. Methadone is addictive.

Q2. Methadone makes me sleepy.

Q3. Methadone decreases my sexual activity.

Q4. Methadone has negative effects on my health (constipation, liver problems, heart pain).

**PC: Perceived concerns.**

Q5. Daily clinic visits for methadone threaten my family life and my job.

Q6. Daily clinic visits for methadone have a negative effect on my family relationships.

Q7. I’m scared to see one of my relatives in a methadone treatment center.

**PPE: Perceived positive effects.**

Q8. Methadone reduces the temptation to consume drugs.

Q9. I’ve had more clean days with methadone.

Q10. Methadone makes me feel euphoric.

**PB: Perceived barriers.**

Q11. Methadone does not help addiction treatment.

Q12. Methadone treatment is long term.

Q13. Methadone does not have an effect on the prevention of relapse.

Q14. Daily clinic visits for methadone are hard for me.

Q15. Methadone treatment centers are not available enough.

Q16. I do not receive adequate counseling at the Methadone Therapy Center.

Q17. Methadone treatment is costly.

## Appendix 2.

Q2. Methadone makes me sleepy.

Q11. Methadone does not help addiction treatment.

Q13. Methadone does not have an effect on the prevention of relapse.

Q15. Methadone treatment centers are not available enough.

## Abbreviations

CFA: Confirmatory factor analysis
CFI: Comparative fit index
CVI: Content validity index
CVR: Content validity ratio
DUAMMT: Drug users’ attitudes toward methadone maintenance treatment
EFA: Exploratory factor analysis
GFI: Goodness of fit index
ICC: Intra-class correlation coefficient
MMT: Methadone maintenance treatment
NFI: Normed fit index
NNFI: Non-normed fit index
RMSEA: Root mean square error of approximation;
SRMR: Standardized root mean square residual
